# Fixed dose combination of low dose pregabalin and duloxetine, or pregabalin monotherapy for neuropathic pain: A double-blind, randomized, parallel-group study

**DOI:** 10.12688/f1000research.130345.1

**Published:** 2023-03-30

**Authors:** Krishnaprasad K., Sunil Dutt, Pankaj Rattan, Ankit Dadhania, Ram Gupta, Deepa Joshi, Ashutosh Kakkad, Altaf Makwana, Pankaj Jha

**Affiliations:** 1 Medical Services, Torrent Pharmaceuticals Limited, Gandhinagar, Gujrat, 382428, India; 2 Torrent Pharmaceuticals Limited, Torrent Research Center, Bhat, Gandhinagar, Gujarat, 382428, India

**Keywords:** Pregabalin, duloxetine, fixed-dose combination, neuropathic, pain, numeric pain rating scale (NPRS)

## Abstract

**Background:** Treatment of neuropathic pain is challenging. Pregabalin and duloxetine are used as first-line therapy. Various international guidelines recommend a combination of first-line agents for the management of neuropathic pain. The objective of this study was to evaluate the efficacy and safety of a fixed-dose combination (FDC) of low-dose pregabalin and duloxetine compared to pregabalin monotherapy at week 7 in patients with moderate to severe neuropathic pain.

**Methods:** This was a phase 3, randomized, double-blind, double-dummy parallel-group non-inferiority study conducted at 17 sites across India. Three hundred and twenty-eight adult patients with moderate to severe neuropathic pain were randomized in a ratio of 1:1 to receive a FDC of pregabalin and duloxetine or pregabalin monotherapy for 7 weeks followed by a one-week follow-up. The pregabalin-duloxetine combination was initiated at 50 plus 20 mg per day and gradually titrated to a maximum of 75mg plus 30mg twice daily. Pregabalin was initiated at 75mg/day and gradually titrated to a maximum of 150mg twice daily. The main efficacy outcome was a mean change in pain intensity at the end of 7 weeks.

**Results:** Two hundred and ninety-eight patients completed the study, 148 in the pregabalin-duloxetine group and 150 in the pregabalin group. The mean change in daily pain at 7 weeks was as follows: -4.49 with FDC and -4.66 with pregabalin (p<0.0001). The non-inferiority of a low-dose FDC compared to pregabalin monotherapy was demonstrated at the end of the study. The incidence of dizziness and somnolence was comparable between both treatments. A higher frequency of peripheral oedema was observed with pregabalin monotherapy than in the FDC group (p>0.05).

**Conclusions:** A FDC of low doses of pregabalin and duloxetine and high dose of pregabalin monotherapy achieved similar analgesia with dizziness, and somnolence as the most frequent adverse event.

**Trial registration:** CTRI/2020/09/027555

## Introduction

Neuropathic pain significantly impacts the quality of life and well-being of patient. ^1^ The financial burden to manage neuropathic pain in the chronic setting is huge on society. ^2^^,^^3^ Its prevalence in the general population ranges from 1% to 8%. ^4^^,^^5^

The most effective pharmacotherapeutic classes of drugs in the management of neuropathic pain are gabapentanoids, tricyclic antidepressants (TCAs) and serotonin-norepinephrine reuptake inhibitors (SNRIs). ^1^^–^^3^^,^^6^^–^^9^ Most of these options are effective but come with a few potential side effects.

Pregabalin, a gabapentanoid blocks the presynaptic alpha 2 delta subunit of calcium channels of the dorsal horn. This leads to inhibition of neurotransmitter release from presynaptic neuron. ^7^^,^^8^^,^^10^ Multiple international societies recommend it as a first-line treatment option for neuropathic pain. ^2^^,^^6^^,^^11^

SNRIs are also recommended as a first-line treatment option by multiple guidelines. ^1^^–^^3^^,^^6^^–^^9^ Duloxetine, an SNRI modulates pain signals by facilitating descending inhibitory pain pathway. ^3^^,^^7^ SNRIs have been found effective in peripheral diabetic neuropathy, ^6^^,^^8^ painful peripheral neuropathy and multiple sclerosis. ^12^

Treatment of neuropathic pain with monotherapy has several limitations such as not being effective for all patients, having partial pain relief and increased side effects with higher doses. ^8^ These are the main reasons to use two or more drugs in about 45% of patients. ^13^^,^^14^ To overcome these issues, for adequate pain relief without increased adverse effects, combination therapy with two or more medications is required in about half of the patients affected with neuropathic pain. Many international guidelines suggest an addition of another drug from a different pharmacological class when adequate pain control is not achieved with monotherapy. However, the evidence available for combination therapy is limited. ^1^^,^^2^^,^^7^^,^^8^^,^^9^ The advantages of combination therapy may be increased efficacy and better tolerability due to the use of smaller doses of individual drugs. ^1^^,^^9^

Both pregabalin and duloxetine are approved in the treatment of neuropathic pain. The monotherapy with drugs at their standard doses (60mg and 300 mg/day respectively) have shown to provide substantial clinical pain relief in only 40% of patients. ^15^^,^^16^^,^^17^

Administration of pregabalin and duloxetine combination treatment may provide better pain relief and tolerability than the administration of maximum doses of each drug, especially in patients showing partial response to monotherapy. ^18^

Pregabalin and duloxetine have different mechanism of action and administration of these drugs in combination rather than monotherapy may potentially provide enhanced pain relief in neuropathic pain due to their complementary actions. ^19^^,^^20^ However, there have been no clinical studies on fixed-dose combinations of low-dose pregabalin and duloxetine for the treatment of neuropathic pain.

The objective of our phase 3 study was to assess the potential non-inferiority of fixed-dose combination of low-dose pregabalin and duloxetine compared to pregabalin monotherapy in adults with moderate to severe neuropathic pain.

## Methods

### Study design and setting

This was a multicentre, parallel, randomized, double-blind, double-dummy, non-inferiority trial conducted in adult patients with neuropathic pain enrolled from 17 sites across India. All investigational sites were approved by respective Independent Ethics Committee or Institutional Review Board before the enrolment of any patient in the trial. This trial followed the principles outlined in the Declaration of Helsinki and the trial protocol was approved by the Institutional Ethics Committee and the Drug Controller General of India (CT-06-11/2020). The trial was registered on 2 ^nd^ September 2020 with the Clinical Trial Registry of India (Registry identifier: CTRI/2020/09/027555). This trial was conducted from September 2020 to January 2021.

### Study population

Four hundred and thirty patients presenting with moderate to severe neuropathic pain and having at least moderate to severe pain intensity ≥ 4 on the numeric pain rating scale (NPRS) during screening at one of the participating centres were assessed and referred to the investigator for enrolment. The pain intensity was measured through 11-point NPRS in which 0 indicated no pain and 10 as the worst possible pain. Eligible adult patients aged 18 to 65 years of age who met inclusion criteria and signed informed consent forms were enrolled. Exclusion criteria included were a history of hypersensitivity to pregabalin or duloxetine or any of its excipients; clinically significant illness in the past three months; type I or II diabetes with glycosylated haemoglobin A1C of >10%; clinically significant electrocardiogram (ECG) abnormalities or QTc ≥450 msec; treated with drugs that impair metabolism of serotonin or serotonergic drugs other than study drugs or corticosteroids within the past four weeks; treated with topical or systemic pain medications within past two weeks; pregnant or lactating women, female with childbearing potential who were neither surgically sterilized nor willing to use medically acceptable contraceptive methods during the study; participated in any other investigational drug trial within the past four weeks and those patients who in the opinion of the investigator either unable to cooperate or unlikely to adhere with any study procedures or not considered a suitable candidate for participation in the study.

### Treatment & visit details

The trial period was 7 weeks, and the patients were scheduled for 7 visits (screening, baseline, week 1, week 2, week 3, week 5 and week 7). Patients were screened by the respective site investigator and eligible patients were randomized to receive either test + dummy reference or reference + dummy treatment. The assignment of treatment was as per the pre-determined computer generated, centrally administered randomization scheduled with the help of interactive web response system (IWRS). Each patient received unique randomization code.

Post-screening, three hundred and twenty-eight participants were randomized to receive either fixed-dose combination (FDC) of pregabalin and duloxetine or pregabalin monotherapy. At screening, sociodemographic data and pain intensity were assessed. At baseline, clinical data (significant medical history, concomitant use of other medications), pain intensity and neuropathic pain symptom inventory were collected.

At the baseline visit, all the eligible patients were randomized in a 1:1 fashion to receive either FDC of pregabalin and duloxetine plus dummy of pregabalin (Test group) or pregabalin plus dummy of FDC of pregabalin and duloxetine (Reference group). The study medications were administered in a double-blind, dummy manner. The double-dummy design of the trial was achieved with the placebo formulations of pregabalin plus duloxetine and pregabalin in identical capsule. The investigation product’s primary packaging, blinding, labelling and secondary packaging was performed by third party. The investigation packages were given unique number code. At regular monitoring visits, study monitor ensured that the blinding was maintained. The participants, the research staff, and the investigators were all blinded to treatment allocations throughout the study period. One-hundred and sixty-four patients in the test group were initiated with FDC of pregabalin 50mg plus duloxetine 20mg capsule and one-hundred and sixty-four patients in the reference group with pregabalin monotherapy (75mg) capsule in the evening. Patients who responded to assigned treatment continued the same treatment until week 7 and those who were non-responders were up titrated after assessment at consecutive weeks. Patients who were having <30% reduction in pain intensity (based on the 11-point NPRS) were considered non-responders to the assigned treatment. After visit 2, non-responders in both groups were up-titrated at visit 3, visit 4 and visit 5 ( Table 1). A telephonic safety follow-up was performed for all the subjects after 1 week (± 2 days) after the end of the treatment.

**Table 1.** Up-titration in Non-responder (<30% reduction in pain intensity based on the 11 point NPRS) patients.

Test Group (FDC Pregabalin + Duloxetine)	Reference Group (Pregabalin)
• Visit 3-75 mg plus 20 mg in the evening • Visit 4-75 mg plus 30 mg in the evening • Visit 5-75 mg plus 30 mg in the morning and evening	• Visit 3-100 mg in the evening • Visit 4-150 mg in the evening • Visit 5-150 mg in the morning and evening

### Study outcome measures

The primary objective was to assess the mean change in pain intensity based on an 11-point NPRS.

The secondary outcome measures were mean change in NPRS from baseline to weeks 2, 3, and 5; proportion of responders (≥30% improvement in pain (NPRS) from baseline) at week 7; proportion of patients with ≥50% improvement in NPRS from baseline to week 7; mean change in NPSI total score from baseline to end of the treatment; Percentage of patients who required rescue medication for inadequate pain relief (recorded at every visit); Patient Global Impression of Improvement (PGI-I) score at week 7; Clinical Global Impression of Improvement (CGI-I) score at week 7; incidence of treatment-emergent adverse event (TEAE) in test arm against reference arm (recorded at every visit)

### Statistical analysis

The study was powered to detect a mean of improvement of 2.5 for Pregabalin and 2.7 for FDC at the end of the study in NPRS with a 2-sided test and 95% power, assuming an SD of 2.3. Non-inferiority margin considered was 33%. This analysis required 139 patients per treatment arm. We assumed around 15% discontinuing during therapy, on that basis a total of 328 patients were planned.

For analyses, we used modified intention-to-treat (mITT) principles. All randomised patients were included in efficacy analyses if they were treated and had a baseline and at least received one dose of study medication and one post-baseline assessment of any efficacy parameter. In safety population analysis, all randomised patients who received at least one dose of study medication were included.

Primary efficacy evaluation, change from baseline (Day 0) to EOT (Week 7) was calculated for NPRS score. It was compared using an independent t-test to test the differences between treatment groups. The non-inferiority was assessed based on the one-sided 97.5% CI (i.e., two-sided 95% CI) approach. A non-inferiority margin of 33% (MoNI -0.8) of the total response was observed for reference.

The secondary efficacy analysis included mean change in NPRS score from baseline to Weeks 2, 3 and 5 and mean change in NPSI total score from baseline to end of week 7, PGI-I and CGI-I score, subjects required rescue medication for inadequate pain relief and incidence of TEAE [dizziness, somnolence and/or peripheral oedema]. These endpoints were analyzed using either t-test, Chi-square or Fisher’s exact test.

Descriptive statistics were used to summarise the safety measures. Fisher’s Exploratory Analysis were performed to determine the frequencies of patients with TEAEs. The analysis of variance (ANOVA) and analysis of covariance (ANCOVA) were performed to determine the difference in the least-squares mean in the magnitude of change in NPRS and NPSI scores between the treatments. All statistical analysis were performed by using SAS software, version 9.4 (SAS Institute Inc., North Carolina, Cary, USA).

## Results

**Figure 1.**
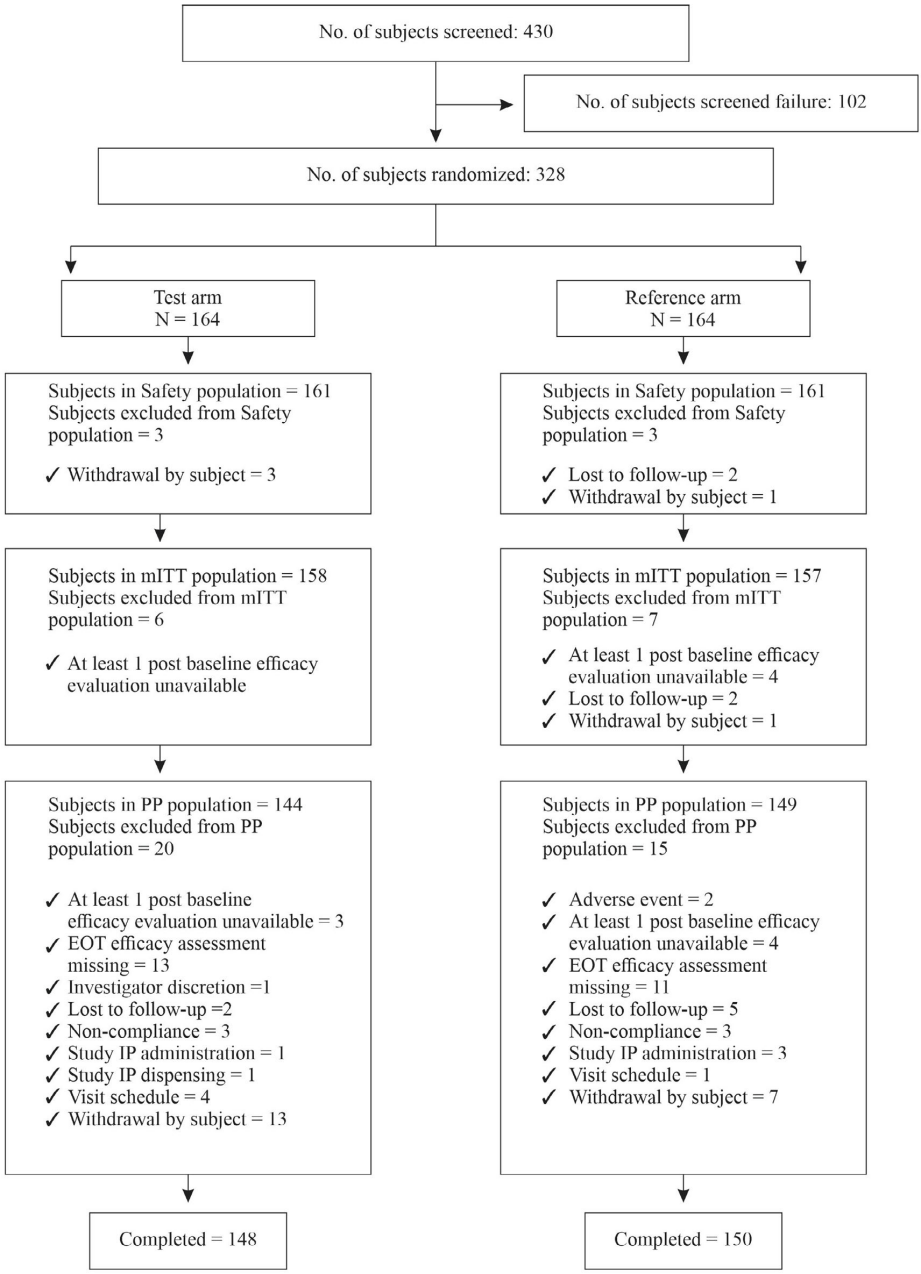
Diagrammatic Representation of Subject Populations.

A total of 430 patients were screened from all the sites ( Figure 1). A total of 328 eligible patients were equally randomized in two groups as test groups (N=164) and reference group (N=164). After randomization, 20 patients withdrew their consent, 7 patients were lost to follow-up, 2 patients discontinued due to adverse events and 1 patient was discontinued by the investigator from the test group. The reasons for discontinuation are mentioned in Figure 1. Thus, total 298 patients completed 7 weeks treatment. ^28^

The demographic characteristics between both treatment groups were comparable. The number of female patients was slightly higher in the reference group compared to the test group. The demographic characteristics are presented in Table 2. The mean patient age at enrolment was 46.5 years. All included patients had an average pain score of at least 4 on NPRS prior to treatment. The mean pain intensity was 6.4.

**Table 2.** Summary of Baseline and Demographics Characteristics - Safety Population.

Parameter/Statistics	Test (N = 161)	Reference (N = 161)	Overall (N = 322)
Age (Completed years)			
n	161	161	322
Mean ± SD	46.1 ± 11.32	47.0 ± 10.84	46.5 ± 11.08
Median	47.0	48.0	48.0
Min, Max	18, 64	23, 64	18, 64
p-value	0.4364		
Sex, n (%)			
Male	92 (57.14)	72 (44.72)	164 (50.93)
Female	69 (42.86)	89 (55.28)	158 (49.07)
p-value	0.0258		
Race, n (%)			
Asian	161 (100)	161 (100)	322 (100)
p-value	NE		
Ethnicity, n (%)			
Not- Hispanic or Latino	161 (100)	161 (100)	322 (100)
p-value	NE		
Height (cm)			
n	161	161	322
Mean ± SD	159.47 ± 9.587	158.85 ± 8.709	159.16 ± 9.149
Median	159.80	158.00	159.00
Min, Max	132.0, 186.0	139.0, 188.0	132.0, 188.0
p-value	0.5481		
Weight (kg)			
n	161	161	322
Mean ± SD	67.300 ± 13.2813	67.820 ± 11.5493	67.560 ± 12.4288
Median	66.900	68.000	67.600
Min, Max	36.00, 127.50	47.00, 106.00	36.00, 127.50
p-value	0.7076		
BMI (kg/m ^2^)			
n	161	161	322
Mean ± SD	26.5 ± 5.00	26.9 ± 4.55	26.7 ± 4.78
Median	26.0	26.0	26.0
Min, Max	15, 44	19, 45	15, 45
p-value	0.4019		
NPRS measurement			
n	161	161	322
Mean ± SD	6.4 ± 1.19	6.5 ± 1.30	6.4 ± 1.25
Median	6.0	6.0	6.0
Min, Max	4, 10	4, 10	4, 10
p-value	0.3968		
NPSI measurement			
n	161	161	322
Mean ± SD	48.7 ± 13.71	49.4 ± 15.48	49.1 ± 14.60
Median	47.0	49.0	48.0
Min, Max	0, 79	6, 78	0, 79
p-value	0.6587		

Treatment Specification: Test = Fixed dose combination of Pregabalin and Duloxetine capsule; Reference = Pregabalin capsule.

Abbreviation(s): BMI = Body Mass Index; cm= Centimeter; kg= Kilogram; m= Meter; Max = Maximum; Min = Minimum; N = Number of subjects in specified treatment; n = Number of subjects in specified category; NE = Not Evaluable; NPRS = Numeric Pain Rating Scale; NPSI = Neuropathic Pain Symptom Inventory; SD = Standard Deviation.

Note 1: Percentages were based on the number of subjects in the specified treatment.

Note 2: For continuous variables p-value was calculated using independent t-test and for categorical variables p-value was calculated using Chi-square test.

Note 3: NPRS and NPSI measurement were based on Visit 2 (Day 0).

Note 4: Safety = Randomized subjects administered with at least one dose of study medication.

### Primary outcomes

**Figure 2.**
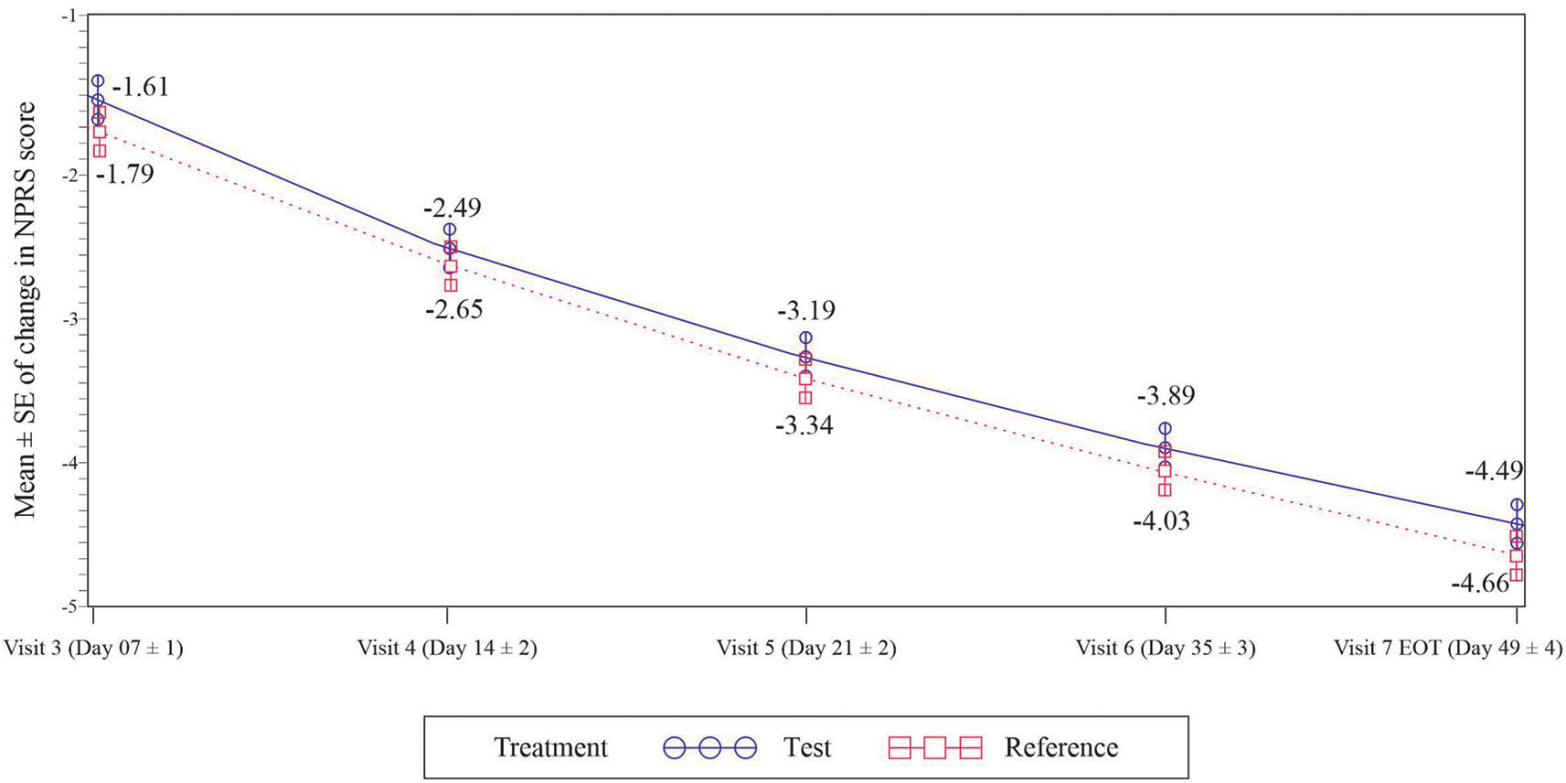
Mean Change from Baseline in NPRS Score at Week, 1,2,3,5 and 7 - mITT population.

The primary efficacy assessment was to compare the change in NPRS score between the test and the reference group. At the end of week 7, the mean change in NPRS score from baseline was -4.49 and -4.66 with the test and the reference group (p<0.0001) respectively ( Figure 2). The lower bound 95% CI for treatment difference was -0.18 which was higher than the non-inferiority margin of -0.8. Therefore, the test treatment was non-inferior to the reference group. These results obtained in the mITT population were supported by those seen in the PP population [95%CI: -0.26, 0.44, p <0.05).

### Secondary outcomes

The change in NPRS scores between the test group and the reference group at weeks 2, 3 and 5 from baseline was non-significant. In the test group it was -2.49, -3.19 and -3.89 while -2.65, -3.34 and -4.03 in the reference group (p>0.05). ( Figure 2)

The percentage proportion of responders between the test and the reference treatment were similar at week 7 (96.20% Vs 98.73%, p>0.05). Similarly, no statistically significant difference was observed at week 7 in the percentage of the patient with ≥50% improvement in pain (84.18% Vs 89.17, p>0.05). No statistically significant difference in mean change in NPSI total score from baseline was observed (-37.57 Vs -38.47, p>0.05).

When patients were asked about their global impression of improvement using the PGI-I, patients reported much better to very much better improvement with both treatments. The mean of the PGI-I score at Week 7 was 1.7 and 1.6 between test group and treatment group (p>0.05).

When investigators were asked about their global impression of improvement using the CGI-I, investigators reported much better to very much better improvement with both the treatments. The mean of CGI-I scores at week 7 was 1.6 in both the treatment groups (p>0.05). Table 3 represents the secondary outcome data.

**Table 3.** Secondary Outcome Parameters.

Parameter	FDC of Pregabalin and Duloxetine	Pregabalin Monotherapy	P Value
Proportion of responders with ≥30% from baseline to week 7	96.20%	98.73%	p>0.05
Proportion of responders with ≥50% from baseline to week 7	84.18%	89.17%	p>0.05
Mean change in NPSI total score from baseline to week 7	-37.57	-38.47	p>0.05
Percentage of patients who required rescue medication for inadequate pain relief	27.95%	29.81%	p>0.05
Patient Global Impression of Improvement (PGI-I)	1.70	1.60	p>0.05
Clinical Global Impression of Improvement (CGI-I)	1.60	1.60	p>0.05
Incidence of treatment emergent adverse event	36.65%	34.78%	p>0.05
Frequency of peripheral edema	0%	3.11%	p>0.05

### Safety

The percentage of subjects who experienced TEAEs were comparable between the groups. Most of the TEAES were at least possibly related to study treatment. In the reference group, two patients discontinued the treatment due to TEAES while none in the test group discontinued for this reason. Table 4 presents an overall summary of adverse events for the safety population.

**Table 4.** Overall Summary of Adverse Event - Safety Population.

Variable	Test (N = 161) n (%) [E]	Reference (N = 161) n (%) [E]	Overall (N = 322) n (%) [E]
Number of subjects with at least one:			
Adverse Event (AE)	59 (36.65) [89]	56 (34.78) [74]	115 (35.71) [163]
Mild	56 (34.78)	50 (31.06)	106 (32.92)
Moderate	4 (2.48)	8 (4.97)	12 (3.73)
Severe	0 (0)	1 (0.62)	1 (0.31)
TEAE	59 (36.65) [88]	56 (34.78) [73]	115 (35.71) [161]
Mild	56 (34.78)	50 (31.06)	106 (32.92)
Moderate	4 (2.48)	8 (4.97)	12 (3.73)
Severe	0 (0)	1 (0.62)	1 (0.31)
Serious Adverse Event (SAE)	0 (0)	0 (0)	0 (0)
Treatment Emergent SAE	0 (0)	0 (0)	0 (0)
Subjects Discontinued due to:			
SAE	0 (0)	0 (0)	0 (0)
TEAE	0 (0)	2 (1.24)	2 (0.62)
Leading to Death	0 (0)	0 (0)	0 (0)

Treatment Specification: Test = Fixed dose combination of Pregabalin and Duloxetine capsule; Reference = Pregabalin capsule.

Abbreviation(s): E = Number of events in specified category; AE = Adverse event; N = Number of subjects in specified treatment; n = Number of subjects in specified category; SAE = Serious adverse event; TEAE = Treatment emergent adverse event.

Note 1: Subjects with multiple events in the same category were counted only once in that category with highest severity.

Note 2: Percentages were based on the number of subjects in safety population in specified treatment.

Note 3: Medical Dictionary: MedDRA Version 23.0.

Note 4: Safety = Randomized subjects administered with at least one dose of study medication.

The major side effects reported in both groups were somnolence, dizziness, and oedema ( Table 4). All the reported TEAEs were mild in severity. The incidence of peripheral oedema was higher with the reference treatment as compared to the test treatment (3.11% vs. 0%, p>0.05), although statistically not significant.

No serious adverse events (SAE) were observed in the study.

In additional analysis, significantly more moderate to severe adverse events were observed in the reference group as compared to the test group. Furthermore, early onset of somnolence and gastrointestinal AEs were observed with pregabalin monotherapy compared to FDC of low-dose pregabalin and duloxetine and this difference was significant. Similarly, significantly, more patients withdrew from the study due to experiencing AE’s or needing treatment for AE’s in the test group compared to the reference group.

## Discussion

Neuropathic pain can be spontaneous or provoked and the patient may complain of paraesthesia, dysesthesia, and loss of normal sensation due to nerve damage. ^21^ The most commonly used option to treat neuropathic pain is pharmacotherapy. However, the complex aetiology of neuropathic pain is the reason for the limited benefit of monotherapy.

The drawback of monotherapy is its limited efficacy and increased adverse effects with the higher dose. Due to tolerability issues, higher doses needed for maximum therapeutic response are not used. Inadequate partial pain relief with the single agent will lead to impaired physical function, impaired sleep, reduced quality of life and higher economic costs of unrelieved pain.

Combination therapy with two or more drugs must be considered to overcome the challenges of managing uncontrolled neuropathic pain with improved analgesic efficacy and reduced overall side effects especially when synergistic interactions are possible for dose reductions of combined drugs. ^22^ In clinical practice, combination therapy is frequently used.

Evidence supports the combination of gabapentinoids and antidepressants than a high dose of individual drugs from either class for a reasonable treatment in neuropathic pain. Pregabalin, a gabapentinoid and duloxetine, an antidepressant; should be initiated with lower doses and gradually up-titrated based on the treatment response.

Pregabalin-duloxetine combination shows additive analgesic actions with a better safety profile compared to high-dose pregabalin monotherapy. In clinical practice, lower doses (once daily or twice daily) of pregabalin (50mg, 75 mg) and duloxetine (20mg, 30 mg) are widely co-prescribed by the neurologist. A combination of significantly lower doses of pregabalin-duloxetine has a greater pain relief than for side effects. ^23^

Danish expert recommendation reported that a combination of gabapentinoid and SNRI is associated with better pain relief and lesser side effects than a single drug therapy. ^24^ An Indian consensus statement suggests that when managing neuropathic pain management with gabapentinoids as first drug, TCAs such as nortriptyline or SNRIs can be considered as add on therapy and vice versa. ^25^ The American Diabetes Asssociation (ADA) position statement recommends combination of two first line agents from class of gabapentinoids, SNRIs, or TCAs in cases wherein monotherapy fails. Furthermore, it also suggests that tramadol/tapentadol can be added if two drug combinations provide inadequate pain relief. ^26^

The COMBO-DN (Combination vs. Monotherapy of pregabalin and duloxetine in DPN) is one of the largest and well known multinational randomized study conducted in patients with diabetic peripheral neuropathy. It reported outcome of duloxetine and pregabalin monotherapy at high doses versus a combination of these two agents at standard dosing. ^27^ The primary outcome of the study was not achieved as no significant difference was measured between the monotherapy and standard-dose combination therapy. However, secondary outcomes of this trial showed that number of patients who achieved >50% reduction were more in combination treatment compared to high dose monotherapy (52.1% Vs 39.3%, p=0.068) with better tolerability.

The initial evidence indicates the need of additional large clinical trials in neuropathic pain for identifying optimal combination as initial approach. Therefore, we conducted a phase 3 trial on the fixed-dose combination of lower doses of pregabalin (50mg, 75mg) and duloxetine (20mg, 30mg) in adult patients with moderate to severe neuropathic pain.

Our phase 3 trial result suggests that the combination of a low dose of pregabalin and duloxetine provides similar analgesia and better safety in comparison to a high dose of pregabalin (150mg, 300mg) monotherapy. The subjective assessment done by CGI-I and PGI-I was also found similar in both the groups.

Data from the present trial suggest that a combination of low-dose pregabalin and duloxetine could be a combination of choice considering the relatively lesser dose administration for pregabalin without compromising efficacy and safety. Additional evaluation of safety data indicated a clear tolerability advantage of FDC over monotherapy for clinically meaningful adverse events associated with monotherapy.

The efficacy results of previous studies on the combination of pregabalin and duloxetine indicate that a combination therapy might be a reasonable therapeutic approach rather than up-titrating the dose in patients with inadequate pain control or having tolerability problem with a high doses of single first-line agent. The outcome of the present study supports the outcomes of previous studies.

In our trial, there were no significant safety concerns observed in both the treatment groups. The most common adverse effects observed were dizziness and somnolence, which are in line with the currently known profile of these drugs.

In this study, the overall frequency of adverse effects was similar with both treatments, except that patient receiving the pregabalin monotherapy had a higher frequency of peripheral oedema than those receiving the pregabalin-duloxetine combination. Additional analysis also established the safety advantage of FDC in AEs of common concern over monotherapy.

The strength of our study is that it is a double-blind, double-dummy parallel-group design. The use of lower doses of pregabalin and duloxetine in combination reflects current clinical practice.

The limitations of our study include small sample size, shorter duration of the study, and the design of the study which compared a fixed dose combination of pregabalin and duloxetine only with a high dose of pregabalin not with duloxetine. Patient related outcomes such as sleep interference and emotional functioning were not assessed in our trial.

Nevertheless, the trial results unequivocally shows that a low dose pregabalin-duloxetine combination is non-inferior to high-dose pregabalin monotherapy in management of patients with moderate to severe neuropathic pain.

## Conclusion

In patients with moderate to severe neuropathic due to different aetiologies, the analgesic effect demonstrated by FDC of pregabalin (low dose), and duloxetine was comparable to pregabalin (high dose) monotherapy. The FDC was safe and well tolerated. This is the first Indian clinical trial in which FDC of pregabalin and duloxetine was evaluated. Future studies with larger sample sizes, long-term duration and heterogenous population are necessary to understand the clinical outcome with FDC of pregabalin and duloxetine.

## Data Availability

Figshare: Fixed dose combination of low dose pregabalin and duloxetine, or pregabalin monotherapy for neuropathic pain: A double-blind, randomized, parallel-group study, https://doi.org/10.6084/m9.figshare.21755288.v4. ^28^ This project contains the following underlying data:
- Diagnosis of Patients_ Listing.xlsx - Patient Demographics.xlsx - Patient Medical History.xlsx Figshare: CONSORT checklist for ‘ *Fixed dose combination of low dose pregabalin and duloxetine, or pregabalin monotherapy for neuropathic pain: A double-blind, randomized, parallel-group study*’, https://doi.org/10.6084/m9.figshare.21755288.v4. ^28^ Data are available under the terms of the Creative Commons Zero “No rights reserved” data waiver (CC0 1.0 Public domain dedication).
