# Supplementary material for: Fixed dose combination of low dose pregabalin and duloxetine, or pregabalin monotherapy for neuropathic pain: A double-blind, randomized, parallel-group study
Source: F1000Res. 2023 Mar 30;12:353. [Version 1] doi: 10.12688/f1000research.130345.1 (PMC11016171; doi:10.12688/f1000research.130345.1)
Supplement: Peer review history

# Reviewer report: Reviewer response for version 1

**DOI:** 10.5256/f1000research.143095.r254301

**Published:** 2024-04-16

**Authors:** Nariman Essmat

**Affiliations:** 1 Semmelweis University, Budapest, Hungary

Subject: Review of Manuscript

 The manuscript titled "Efficacy and Safety of Fixed-Dose Combination of Pregabalin and Duloxetine for Neuropathic Pain" provides valuable insights into the efficacy and safety of a fixed-dose combination (FDC) of pregabalin and duloxetine compared to pregabalin monotherapy for the management of neuropathic pain. The manuscript provides a clear overview of the study's objectives, methods, results, discussion, and conclusions. However, the paper needs minor revision before acceptance.

 My comments have been addressed as follows:

- The title does not specify the phase of the study. Including information about the study phase (e.g., phase 3) would be better
- In the abstract: in the method section the authors should clarify the method of pain assessment (numeric pain rating scale (NPRS).
- In the first paragraph of the introduction section, authors should define neuropathic pain according to IASP.
- In the Study Design and Setting section, please mention that the study is in phase III
- Please add more information for the inclusion criteria
- In the statistical analysis section, please explain how the Non-Inferiority Margin was determined.
- DPN: give the full name for this abbreviation, no need for an abbreviation as it appears one in the text
- The study was double-dummy; no data was added for the placebo group; please provide supplementary data on this issue
- In the Primary outcomes section, what does the PP population refer to?
- In Table 3, what was the statistical test used for the calculation of the p-value? It’s better to add a table ligand
- In the conclusion section, it mentioned (In patients with moderate to severe neuropathic due to different aetiologies), please specify the neuropathic pain types that can be treated with this combination

Is the work clearly and accurately presented and does it cite the current literature?

Yes

If applicable, is the statistical analysis and its interpretation appropriate?

Yes

Are all the source data underlying the results available to ensure full reproducibility?

Yes

Is the study design appropriate and is the work technically sound?

Yes

Are the conclusions drawn adequately supported by the results?

Yes

Are sufficient details of methods and analysis provided to allow replication by others?

Yes

Reviewer Expertise:

Pain

I confirm that I have read this submission and believe that I have an appropriate level of expertise to confirm that it is of an acceptable scientific standard, however I have significant reservations, as outlined above.

# Reviewer report: Reviewer response for version 1

**DOI:** 10.5256/f1000research.143095.r254315

**Published:** 2024-04-13

**Authors:** Laith Thamer Al-Ameri

**Affiliations:** 1 Al-Kindy College of Medicine, University of Baghdad, Baghdad, Baghdad, Iraq

Dear Editor, Authors, and Readers,

 A well-written manuscript and good work, such a trial will add much to what is known and previously written about neuropathic pain. Pregabalin is well known to be equivalent to or superior to many other medications in neuropathic pain management, and additional information is needed about the benefits of and combinations with other medications regarding efficacy and tolerability.

 It's worth noting that the manuscript demonstrates strong enrolment criteria, a robust methodology, and the use of appropriate statistical methods, which contribute to the overall quality of the study.

 However, the conclusion section needs to be rewritten to represent the results and be more informative. The authors have to mention the result regarding the efficacy and tolerability of both groups (e.g. **, a low-dose pregabalin-duloxetine combination is non-inferior to high-dose pregabalin monotherapy in the management of patients with moderate to severe neuropathic pain**), a statement already mentioned at the end of the discussion.

Is the work clearly and accurately presented and does it cite the current literature?

Yes

If applicable, is the statistical analysis and its interpretation appropriate?

Yes

Are all the source data underlying the results available to ensure full reproducibility?

Yes

Is the study design appropriate and is the work technically sound?

Yes

Are the conclusions drawn adequately supported by the results?

Yes

Are sufficient details of methods and analysis provided to allow replication by others?

Yes

Reviewer Expertise:

Neurosurgery, Neuroanatomy, Clinical Trials, sleep disorders, neuropathy, and Artificial Intelligence in medical fields

I confirm that I have read this submission and believe that I have an appropriate level of expertise to confirm that it is of an acceptable scientific standard.

## References

[1] : Pregabalin versus Gabapentin Efficacy in the Management of Neuropathic Pain Associated with Failed Back Surgery Syndrome. *J Korean Neurosurg Soc* .2024;67(2) : 10.3340/jkns.2022.0225 202-208 10.3340/jkns.2022.0225 37709550 PMC10924903

# Reviewer report: Reviewer response for version 1

**DOI:** 10.5256/f1000research.143095.r236036

**Published:** 2024-02-11

**Authors:** Denis Dupoiron

**Affiliations:** 1 Anesthesiology and Pain Department, Institut de Cancérologie de l'Ouest, Angers, France

Very good paper, the topic is attractive, the comparative study is well built, and the methodology does not present significant issue.

 The outcomes are well presented and relevant. The results validate observations in clinical practice.

 Moreover, the discussion is helpful and well conducted.

 My only one comment is about limitations, I do think the number of patients included is low.

Is the work clearly and accurately presented and does it cite the current literature?

Yes

If applicable, is the statistical analysis and its interpretation appropriate?

I cannot comment. A qualified statistician is required.

Are all the source data underlying the results available to ensure full reproducibility?

Yes

Is the study design appropriate and is the work technically sound?

Yes

Are the conclusions drawn adequately supported by the results?

Yes

Are sufficient details of methods and analysis provided to allow replication by others?

Yes

Reviewer Expertise:

neuropathic cancer pain

I confirm that I have read this submission and believe that I have an appropriate level of expertise to confirm that it is of an acceptable scientific standard.
